# The Economics of Nursing

**Published:** 1918-05-04

**Authors:** 


### BETTER CONDITIONS OF SERVICE IMPERATIVE.

### The Economics of Nursing.

THE final test of value in employment is emolument. This is a general statement, to which there are notable exceptions, especially in war conditions. Nevertheless, it cannot be denied that all unions, trade and professional, are designed mainly to ensure better conditions of service, and whether these take the form of higher financial reward, shorter hours of labour, longer holidays, increased privileges, pensions, or extended fields of service, they may all be classed under the general head of emolument. There is probably no class of skilled workers in the kingdom where emolument in the wide sense has been so neglected as that of nurses. The nurse begins late; she ceases early; her duties are arduous, her hours of labour long. She receives a low remuneration, and she enjoys no pension prospects. This also is a general statement to which there are exceptions, but in the nurse's case especially the exception proves the rule.

### NO ADEQUATE STANDARD.

Take the Army. The Army is a badly paid profession. A small grocer often enjoys a bigger income than the colonel of a regiment. But the Army is regarded generally as the blue ribbon of the nursing profession, the reason being that ordinarily the hours of duty are reasonable, the pay relatively sufficient, and a pension is assured. Now, if the Army is not over-liberal with its officers, it cannot be assumed that it is over-liberal with its nurses. The only reasonable inference, therefore, is that the Army pay and conditions of service for nurses still constitute no adequate standard of what ought to obtain elsewhere. It happens, of course, that nurses largely are employed in charitable institutions, supported mainly by voluntary contributions, and hence the public have by long usage come unanimously to acquiesce that the nurse ought to be a contributor to the charity. It has not been a question of whether the nurse deserves better, but rather of "Where is the money to come from?"—and the result too often has been that the money does not come from anywhere. The nurse has gone without. This is obviously unsound economics. The story is told of a charitably inclined lady who went to a bootmaker, and said, "Bootmaker, I want you to make a pair of boots, and as they are to be given away in charity I want you to make them cheap." The bootmaker replied, "Madam, I do my own charity!" A nurse ought to be her own mistress where she is charitably disposed, and her services ought not to be availed of where she is out for a livelihood and a career, save on strict market conditions. Why should the humblest hospital worker be paid the current wages of her class, and the hospital food contractor at market prices, while the nurse is expected to work according to a scale regulated by the income of the institution? A trained nurse of the best is a highly skilled worker, and the effectual discharge of her duties involves a high moral standard. Nevertheless she gives her services, and she is expected to give them, on terms which do not obtain in any other field of labour. Adequate account is too seldom taken of the cost and sacrifice of training, of the age when a nurse is fully trained, or of the age when she retires from active service.

### THE ULTIMATE ISSUE.

This is not a sentimental discussion, but one of pure business; and in the long run business considerations become paramount. It may be an easy matter to relegate such considerations to oblivion. It may not be convenient or politic to discuss them, but such matters have a way of settling themselves. Nursing is pre-eminently woman's work, and, from a woman's standpoint, attractive work; but this is a material age, and the modern woman worker is not blind to her prospects. Already there are signs that, after the war especially, she will elect to follow occupations which offer material reward. She may make no outcry, but she may avoid the profession that she might otherwise select if she finds it lacking in emolument. The inevitable result will be a lowering of the standard. There is no avoiding this ultimate issue. It is natural law as invincible as the law of gravitation. Many now in harness will, doubtless, labour on. They see no alternative. But the younger generation, as matters are to-day, will look elsewhere. Then the nursing profession may come to be recruited from the leavings, and no Act of Parliament could provide a remedy.

It is desirable in any economic discussion to arrive at a sound and scientific conclusion. Given a high standard of requirement, the reward ought to be commensurate. The tide of war has borne the nurse to the crest of the wave. Her valuable services have won the appreciation and the gratitude of the British public, and full advantage ought to be taken of this opportunity to establish her employment on a sound economic basis. Provision for old age and infirmity is made, say, for the telephone operator. Why should the trained nurse's prospects of such provision be inferior to her's and most women workers?

### UNITED RANKS AND A CLEAR GOAL.

In order to provide an effectual remedy, British nurses must articulate. They must rally to the standard of union, not in units but in battalions. If they do, they will find a champion, and they will find, moreover, a sympathetic public ready to respond to the call. Their field of labour will extend, the standard of qualification will rise, their lives will be brighter, and the innate dread of having to end their days in poverty and embarrassment will disappear. But let it be clearly understood that their salvation is in their own hands, for no spasmodic advocacy can take the place of unified ranks with a goal clearly defined. Granted these conditions, the time must speedily arrive when the trained British nurse may legitimately and confidently hope to have a place in the sun. IERNE.

